# Extending Barrett’s esophagus cancer risk profile towards genetic abnormalities

**DOI:** 10.1186/1755-8166-6-10

**Published:** 2013-03-04

**Authors:** Reza Asari, Martin Riegler, Sebastian F Schoppmann

**Affiliations:** 1 University Clinic of Surgery, Medical University Vienna, Vienna General Hospital, Waehringer Guertel 18-20, Vienna, Austria

## 

Dear Editor,

With interest we read the article by Bajpai *et al.*[1], entitled “ *Prolonged exposure to acid and bile induces chromosome abnormalities that precede malignant transformation of benign Barrett’s epithelium*”, which has been published in the recent issue of *Molecular Cytogenetics*. Barrett’s esophagus results from gastroesophageal reflux and harbors an increased risk for the development of esophageal adenocarcinoma (0.5% annual cancer risk) [2,3]. Using a fascinating *in vitro* approach the authors modeled the effect of gastroesophageal reflux on immortalized Barrett’s esophagus cells [1]. Bajpai *et al.* found that intermittent exposure of BAR-T cells to acid and bile for 18 to 78 weeks caused a spectrum of genetic abnormalities typical for cancer development, including polyploidy, loss of chromosomes and the development of transformed clones. In addition, the genetic changes evoked by acid and bile exposure format the target protein receptors for tumor stimulating growth factors, i.e. epidermal growth factor (EGF), which are known to promote the growth of gastrointestinal cancers [4]. In contrast, unexposed cells did not exhibit these abnormalities. Remains to be questioned, if the striking observations made by the authors may be of clinical relevance for the diagnosis and the therapy of Barrett’s esophagus?

Conceptually, Barrett’s esophagus results as the consequence of a complex neurohumoral response of the esophageal mucosa to gastroesophageal reflux including acid and bile [2,3]. Thus, by theory, the removal of Barrett’s tissue and the elimination of reflux should contribute to cancer prevention. Going in line with with this notion, recent studies found that elimination of reflux by effective antireflux surgery contributes to increase the yield of radiofrequency ablation to eradicate *Barrett’s esophagus*, when compared to ablation and post-ablational proton pump inhibitor (PPI) therapy, which solely changes the pH of the reflux, but not the reflux *per se*[3,5].

Given that the genetic abnormalities assessed by Bajpai *et al.*[1] can be visualized by modern endoscopic in vivo staining techniques, this may enable us to detect tissue at risk for cancer development [4,6,7] and specifically target our therapies (i.e. ablation, endoscopic resection) towards these areas [3]. Therefore a risk profile- (anamnestic, demographic & endoscopic, esophageal function tests characteristics) [3] and endoscopically visualized genetic profile- based tailored management of Barrett’s esophagus for cancer prevention may be realistic in the future [1,4,6,7]. Thus, Barrett’s positive individuals with increased reflux due to severely impaired function of the esophagus may be offered elimination of acid and bile reflux by an effective anti reflux surgery prior to ablation [3,5]. In contrast, ablation and subsequent PPI therapy seems reasonable for those with Barrett’s esophagus and normal esophageal function and reflux monitoring [3]. Endoscopic radiofrequency ablation would then be targeted to in vivo staining positive genetic abnormal areas [1,6,7]. Taken together, time seems ready to design prospective clinical trials to assess the impact of the above mentioned biological markers for cancer prevention. The authors are kindly asked to comment on the above considerations.

Sincerely,

Reza Asari, Martin Riegler, Sebastian F. Schoppmann.
